# Congenital Stricture of Urethra; Successful Operation

**Published:** 1887-03

**Authors:** W. G. Jameson

**Affiliations:** Rusk Texas


DANIEL'S
### Texas Medical Journal.

PUBLISHED MONTHLY.—SUBSCRIPTION $2.00 A YEAR.

Vol. 2. AUSTIN, MARCH, 1887. No. 9.

### ORIGINAL ARTICLES.

CONTRIBUTED EXCLUSIVELY TO THIS JOURNAL.

*The Articles in this Department are accepted and published with the understanding that we are not responsible for, nor do we indorse the views and opinions of the writers by so doing.*

### CONGENITAL STRICTURE OF URETHRA; THREE IN NUMBER.

READ BEFORE EAST TEXAS MEDICAL ASSOCIATION IN JANUARY, 1887.

*By W. G. Jameson, M. D., Rusk Texas.*

For Daniel's Texas Medical Journal.

CHAS. F., aged ten years, was brought to my office August 15th, 1884, for examination in regard to urethral trouble. The history gathered from the father was, the boy had never been able to pass but a "spray-like stream" from birth, and that he had been troubled more or less with incontinence, which was very annoying at night, frequently soiling the bedding. He had grown much worse within the last year. The general appearance of the boy was very good; seemed a little nervous, but I attributed that to our presence and the horror little fellows have for doctors. He had always enjoyed good health, "save occasionally" would have chills and fever; his home being in malarious district. The impediment to flow of urine had never given any symptoms other than slight cystitis, from which he was suffering; there was also urethretis. The external appearance of penis was normal. We made manual examination of urethra, externally from glans penis to peno-scrotal junction, could feel natural depression on under surface of penis, except at strictured points where it had a corded feel. The external orifice was larger than normal. Within the canal a few lines we saw what we thought was the trouble. A stricture which gave to the orifice a "funnel shape." We were struck with the abnormal condition, knowing that to be normally the most constricted part of the urethra. In order to test character of stream, we had him urinate, which he did with some effort, and to our astonishment the stream passed was just as had been described by the father, instead of falling to the ground in continuous stream, passed like a stream from an atomizer. We tried to pass a filiform bougie, but failed. The mere presence of instruments in urethra caused spasms of stricture, which could not be overcome by constant gentle pressure. We put him under influence of chloroform, trying again to pass a small bougie which we did a short distance; meeting with obstruction, withdrew bougie, and divided first stricture with narrow bistoury; but little hemorrhage followed. I then easily pushed a good sized bougie through to second stricture, thinking that the obstruction offered was an enlarged orifice of follicles situated on floor of urethra. But such was not the case. I soon knew that I had to contend with another stricture. This one being too deep seated to use knife, and having in my pocket-case a small exploring trocar, I gathered the glans between forefinger and thumb, put penis slightly on the stretch, introduced trocar, being careful to direct the point in the exact course of urethra before using any force; I withdrew point of trocar within canula, passed to second stricture where canula was arrested. After assuring myself that it was a stricture, I pressed the trocar out of canula on through stricture. Again drawing trocar within canula and using it as a bougie, I passed on in the direction of bladder. When one inch from second stricture, I met with a third, which was treated in same manner as the second. The canula passed easily again some distance. I withdrew instrument and used bougie, which, to our great relief passed readily through all three strictures on into bladder. Not being satisfied with that, we passed a larger bougie, and let it stay a short time so as to dilate well the constricted parts. I would state that after passing strictures, the urethra between them seemed to be rather dilated,

"pocket like." After he came from under influence of anæsthic, I had him to urinate; he making a good bold stream, which I assure you was not only a great pleasure to the boy, but to the surgeons. It was the first that he had freely passed in his life. I saw him next day and passed bougie, which gave him much pain. Finding that to use it again I would have to resort to the use of chloroform, I desisted, trusting to frequent calls to urinate to keep canal dilated, hoping if any adhesion should form, that they would be broken up by urine dilating urethra. But not knowing whether it would act as thus imagined, I resolved to trust to it rather than use an anæsthetic. I am happy to say, my trust was not in vain. The urine acted like a charm in keeping canal dilated; the result was perfect. I treated the patient some time after operation, for nocturnal incontinence. I saw him a few months ago; his stream was good, in fact as large as that of any boy of his age; hence I may, without vanity, flatter myself on the complete success of the operation, and that too, by means, not exactly laid down in the books, but extemporized to meet the case.

